# Optical glucose sensors based on hexagonally-packed 2.5-dimensional photonic concavities imprinted in phenylboronic acid functionalized hydrogel films

**DOI:** 10.1039/c7ra11184c

**Published:** 2017-11-23

**Authors:** Magdalena Bajgrowicz-Cieslak, Yousef Alqurashi, Mohamed Ismail Elshereif, Ali K. Yetisen, Muhammad Umair Hassan, Haider Butt

**Affiliations:** a School of Engineering, University of Birmingham, Edgbaston, Birmingham B15 2TT, UK. Email: h.butt@bham.ac.uk; Tel: +44 (0)121 4158623; b Harvard Medical School and Wellman Center for Photomedicine, Massachusetts General Hospital, 65 Landsdowne Street, Cambridge, MA 02139, USA; c Centre for Micro and Nano Devices, COMSATS Institute of Information Technology, Park Road, Islamabad, 44000, Pakistan

## Abstract

Continuous glucose monitoring aims to achieve accurate control of blood glucose concentration to prevent hypo/hyperglycaemia in diabetic patients. Hydrogel-based systems have emerged as a reusable sensing platform to quantify biomarkers in high-risk patients at clinical and point-of-care settings. The capability to integrate hydrogel-based systems with optical transducers will provide quantitative and colorimetric measurements *via* spectrophotometric analyses of biomarkers. Here, we created an imprinting method to rapidly produce 2.5D photonic concavities in phenylboronic acid functionalized hydrogel films. Our method exploited diffraction properties of hexagonally-packed 2.5D photonic microscale concavities having a lattice spacing of 3.3 μm. Illumination of the 2.5D hexagonally-packed structure with a monochromatic light source in transmission mode allowed reversible and quantitative measurements of variation in the glucose concentration based on first order lattice interspace tracking. Reversible covalent phenylboronic acid coupling with *cis*-diols of glucose molecules expanded the hydrogel matrix by ∼2% and 34% in the presence of glucose concentrations of 1 mM and 200 mM, respectively. A Donnan osmotic pressure induced volumetric expansion of the hydrogel matrix due to increasing glucose concentrations (1–200 mM), resulted in a nanoscale modulation of the lattice interspace, and shifted the diffraction angle (∼45° to 36°) as well as the interspacing between the 1^st^ order diffraction spots (∼8 to 3 mm). The sensor exhibited a maximum lattice spacing diffraction shift within a response time of 15 min in a reversible manner. The developed 2.5D photonic sensors may have application in medical point-of-care diagnostics, implantable chips, and wearable continuous glucose monitoring devices.

## Abstract 2

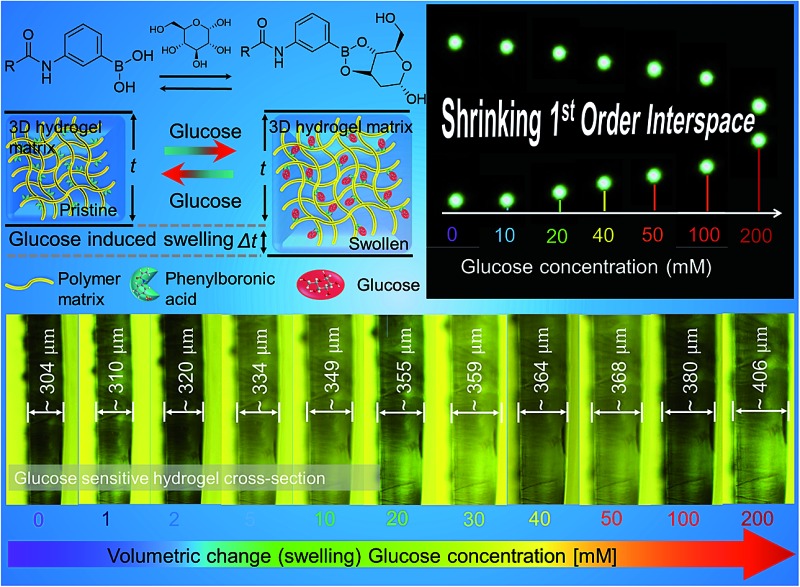
A glucose-responsive hydrogel that changes its volume when exposed to different glucose concentrations was used to measure the glucose levels under physiological conditions.

## 1. Introduction

Diabetes is one of the most serious health problems worldwide.^1^,^2^ It is a chronic disease characterized by disorder of glucose metabolism which is reflected in the elevated concentration of blood glucose.^3^,^4^ Health complications caused by diabetes include heart disease, kidney failure, blindness and increase in the disability-adjusted life years.^5^–^7^ In 2015, the estimated diabetes prevalence was 415 million adults, which is projected to reach 642 million by 2040.^8^ This epidemic also poses an enormous economic burden on society;^9^ the direct annual cost of diabetes to the world is more than $827 billion.^10^ Appropriate medication and glucose concentration control can improve treatment efficacy by mitigating the symptoms and reducing the complications.^5^,^11^–^13^ For this reason, glucose monitoring is crucial in diabetes management.

Currently, the most common method of monitoring glucose concentration is the finger prick test which is an electrochemical method based on enzymes such as glucose oxidase, glucose dehydrogenase.^14^ This procedure is inconvenient for patients, and due is invasive, may lead to infections. Additionally, it does not allow real-time measurements and sensors cannot be reused, due to the irreversibility of reactions.^15^ Moreover, the sensitivity of such electrochemical and enzymatic sensors is affected by numerous factors such as interference from the high partial pressure of oxygen, maltose and haematocrit.^14^,^15^ Hence, development of new continuous and non-invasive glucose monitoring system is necessary to overcome problems related to the conventional electrochemical method.^16^ It is highly desirable that the new system would provide information about real-time fluctuations in blood glucose concentrations, which improves the accuracy of insulin administration in diabetes management.^17^ To date, different approaches have been investigated to achieve a complete solution.^18^–^21^ Optical sensors seem to overcome the limitation of existing sensors since they can provide fast, quantitative, measurements in real-time and in a reversible manner.^16^,^22^

Recent advances in photonics and polymer chemistry have enabled the fabrication of photonic sensors on soft hydrogel materials and have led to an increased interest in hydrogel-based optical glucose sensors.^15^ Hydrogels are highly water-absorbing polymers capable of undergoing reversible volume changes.^23^ They can be designed to respond to certain stimuli (*e.g.* temperature, pH, ionic strength, metal ions, antigens, proteins).^24^–^30^ The selectivity is obtained by functionalizing hydrogels with receptor molecules that are sensitive to a particular stimulus or a molecule.^31^,^32^ One promising approach for glucose detection using hydrogels is the covalent incorporation of boronic acids in a copolymer matrix.^33^–^37^ Boronic acids bind to diol-containing carbohydrate species, such as glucose, through a reversible boronate formation.^38^,^39^ Upon binding of boronic acid copolymer with glucose, the polymer network swells and alters its physical and optical properties, which can be used for glucose quantitative analyses.^31^,^32^ Glucose-responsive hydrogels can be incorporated into photonic devices. The inclusion of the photonic sensor into the hydrogel can help in the development of superior analytical devices. Such photonic devices work through controlling and manipulation of the propagation of light.^40^ Over the last two decades, many approaches including laser writing, self-assembly, and layer-by-layer deposition have been demonstrated to create Bragg diffraction gratings, micro-lenses, etalons and plasmonic structures in hydrogels. Although no commercial device has been released yet due to unsatisfactory sensitivity and specificity issues.^10^

**Fig. 1:**
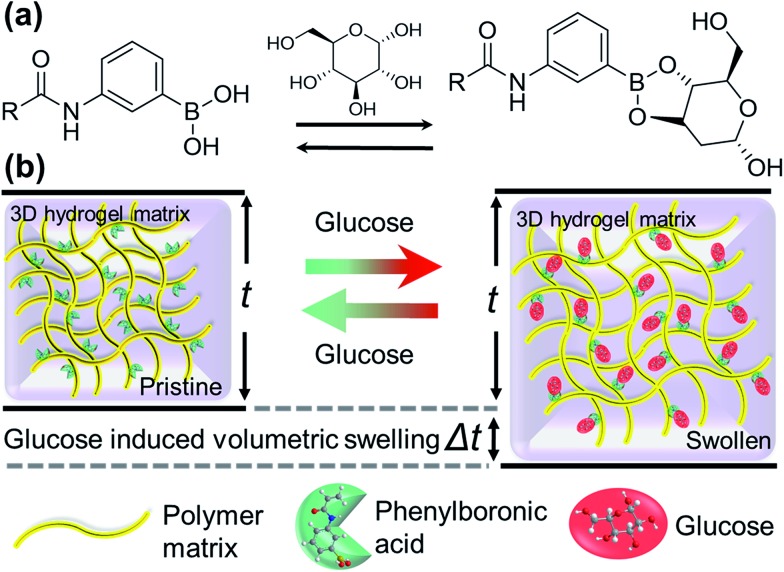
Swelling of the polyacrylamide hydrogel functionalized with 3-(acrylamido)phenyl-boronic acid, induced by the presence of glucose: (a) representation of the reversible chemical attachment of glucose at OH sites of phenylboronic acid, and (b) illustration of such reversible reaction that results in a volumetric change in the boronic acid functionalized hydrogel upon glucose intake or depletion, respectively. Such volumetric modulation can be exploited for glucose sensing.

In this paper, we have proposed a new optical glucose sensor based on a hexagonal diffraction grating imprinted on a flexible hydrogel. The fabrication method is quick and cost-effective. The sensor detected the changes (of overall ∼8°) in the diffraction angle within 15 min due to the increasing glucose concentrations (1–200 mM), see Fig. 1 for the schematic illustration of the concept. This change could also be detected clearly under an optical microscope – the minimum increase in the thickness of the hydrogel sensor was ∼2% for the lowest concentration of 1 mM. These 2.5D glucose sensors could be used multiple times as the detection was observed to be reversible as well as repeatable.

## 2. Results and discussion

**Fig. 2:**
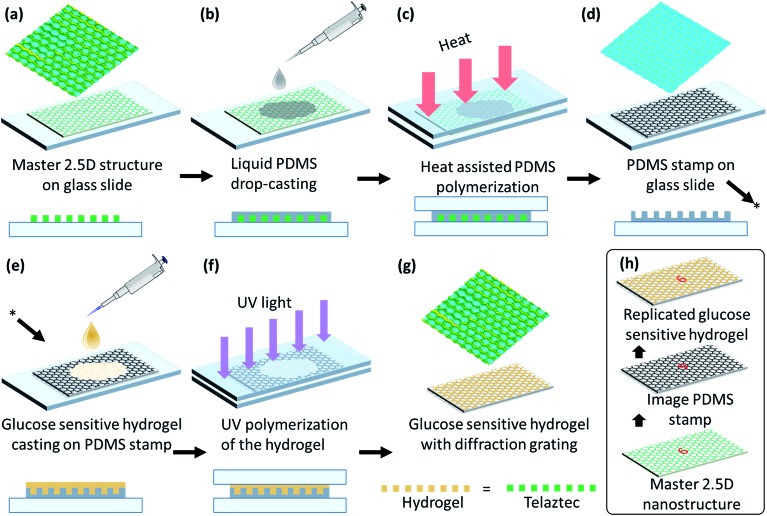
Schematic illustration of imprinting the micrograting on the glucose sensitive hydrogel: (a–d) preparation of PDMS stamp, (e–g) copying the structure from PDMS stamp on a glucose sensitive material, and (h) summary of the replication process.

A honeycomb 2.5D structure was mirror-replicated to obtain a polydimethylsiloxane (PDMS) stamp by a micro imprinting process using a honeycomb master grating.^41^ The PDMS solution was prepared by mixing the PDMS base Sylgards 184 (Dow Corning) with the provided curing agent in a 10 : 1 (w/w) ratio and stirring the solution for 10 min at 24 °C. This solution was placed in low vacuum for 5 min to remove bubbles. The mixture was then poured on the master grating and covered with a glass slide. The sample was cured in an oven for 40 min at 60 °C. The curing process solidified the PDMS, giving a mirror-replica of the parent 2.5D microstructure of the master grating for the subsequent fabrication process of the sensor, see Fig. 2(a–d). The micro-replication process did not damage the original 2.5D grating, such that multiple PDMS stamps could be fabricated from a single master. Subsequently, each individual PDMS stamp could be used multiple times for the preparation of glucose sensors before it starts showing some degrading.

Acrylamide, *N*,*N*′-methylenebisacrylamide, 3-(acrylamido)phenylboronic acid (PBA), dimethyl sulfoxide (DMSO) and 2,2-dimethoxy-2-phenylacetophenone (DMPA) were used as core components of our glucose sensitive hydrogel (GSH): acrylamide (78.5 mol%), *N*,*N*′-methylenebisacrylamide (1.5 mol%) and (PBA) (20 mol%) were mixed together. A solution of 2% (w/v) DMPA in DMSO was added to the mixture at a ratio 1 : 1 (v/v). Subsequently, this mixture was stirred very well (120 min, at room temperature) in order to ensure good homogeneity. The resulting mixture was poured directly onto the PDMS stamp and covered with a glass slide. The thickness of the sample was controlled by controlling the space gap between glass slides by placing a fine shim of a known thickness. The sample was then moved to an ultraviolet (UV) curing chamber and cured with UV light for 5 min. Then, it was kept in DI water for 5 min and peeled off – hydrophobic nature of the surface of the PDMS stamps facilitates an easily peeling off process. Mirror-replication of the 3D structure copied from the PDMS stamp onto the GSH results in copying of the original structure of the hexagonal 2.5D master grating, see Fig. 2(h). All samples were hydrated overnight in deionized (DI) water before further use.

**Fig. 3:**
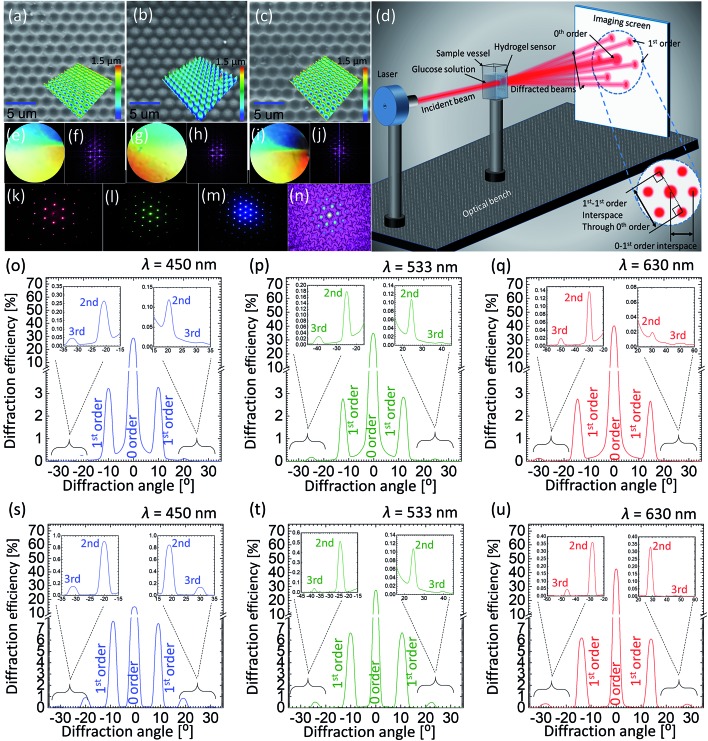
Characteristics of the original and copied honeycomb microstructures: (a–c) SEM images of the 2.5D master structure, PDMS stamp, and glucose sensitive sample, (d) schematic illustration of optical measurement setup, (e–j) photographs taken in white-light for the surfaces of 2.5D sample, PDMS stamp and glucose sensitive sample and theoretical picture of their Fourier transforms, respectively, (k–m) diffraction patterns generated by red, green and blue laser light transmitted through the original glucose sensitive sample, (n) reconstruction of the glucose sensitive sample image by taking Fourier transform of the pattern itself obtained from the patterned hydrogel, (o–q) angle-resolved intensity graphs representing diffraction patterns generated by the 2.5D sample illuminated by the light of blue, green and red laser, (r–t) angle-resolved intensity graphs representing diffraction patterns generated by PDMS stamp illuminated by the light of blue, green and red laser.

The surface of 2.5D grating, PDMS and GSH were imaged by a scanning electron microscope (SEM) (JCM-6000PLUS NeoScope Benchtop). Before imaging, samples were coated with a gold layer (5–10 nm) using Agar sputter coater, to avoid charging effects – specimens being highly dielectric in nature result in charge accumulation and subsequently poor resolution. SEM images show that the hexagonal structure of the 2.5D mimics the true honeycomb architecture, such that the pits with certain depth covered with elevated walls around them form hexagonal cells, with an average cell constant of ∼3.0 ± 0.3 μm and depth (/height) of ∼1.2 μm. The mirror replication of this structure on PDMS is a conjugate fit, *i.e.* domes replace the pits in the mirror-replication process, and the walls in the original structure are now the deeper parts of the replica. The GHS copied from the PDMS stamp again results in the original 2.5D honeycomb (hexagonal) structure, see Fig. 3(a–c). All three specimens exhibit perfect surface morphology with almost no defects suggesting a perfect copying from the 2.5D grating to the PDMS stamp and subsequently from the stamp to the GHS sensor.

The volumetric change of the GSH in the presence of glucose is also one way of measuring the glucose content in solutions, optical microscopy (Axio scope A1, Zeiss) was performed in order to determine the thickness of the pristine sample and in different conditions (after exposing to different glucose concentrations, discussed latter). We obtained the cross-section thickness of ∼221 μm for a dry pristine GSH sensor.

Angle-resolved far-field diffraction experiment were carried out using original 2.5D grating, PDMS and GSH samples, see Fig. 3(d) for schematic illustration of the experiment. The sample was carefully placed in a transparent plastic cuvette, mounted on a motorized precision rotation stage and aligned normal to the incident laser beam. The intensity of each diffracted beam was measured using an image-screen place at a distance of 45 cm away from the sample, as well as, by using an optical power meter (Newport, 1918-R) traversable on a circular rail (CR) of radius of 13 cm with sample mount on its centre (the radius of CR also defines the measurement distance between the sample and the power meter). Three laser sources, red, green and blue (640, 532 and 491 nm) (Newport) were used in diffraction experiments. Measurements were recorded in dry (pristine) and soaked conditions (in PBS solution). In order to perform glucose sensing, the cuvette was filled with different solutions and the whole sample was submerged before taking the measurement. The forward-scattered spectra were collected in all cases either by rotating the cuvette or the detector itself by an increment of 1°, from 0° to 180°, relative to the sample normal. For the reference, the intensity/power of the incident light (blank) was also recorded and percentage of the diffracted intensity for each diffraction spot was calculated. A simple method was adopted to record the glucose induced shift in the spectra: the change in the displacement between two opposite 1st order points such that the displacement line should pass through the centre (0-order) of the diffraction pattern was recorded as a function of glucose concentration. Photographs of the diffracted spectra taken on an imaging screen were also analysed with ImageJ software, and diffraction efficiency (intensity) was plotted against 1^st^–1^st^ order interspace and diffraction angle.

Photographs of the master grating, PDMS and GSH took in white light revealed their diffractive properties as colors present in the incident light were resolved over space, see Fig. 3(e–i) for photographs of all three samples along with computationally calculated Fourier transforms (FT) of their microscopic images revealing their hexagonal architecture. Fig. 3(k–m) shows an example of experimentally obtained diffraction from the PDMS stamp, whereas a reverse FT can be exploited to redraw the physical structure where the light originally diffracts from. We plotted angle-resolved diffracted intensities normalize (to 1) for up to 3^rd^ diffraction orders as the function the diffraction angle for the original 2.5D grating and PDMS stamp in Fig. 3(o–t). The 0-order peak was the strongest in both cases suggesting that most of the light was transmitted straight to the 0 order without being diffracted: blue, green and red illumination resulted in 0-order intensities of 29, 35 and 40% for 2.5D grating, and 16, 28 and 41% for the PDMS stamp, respectively. Intensities of increasing orders (1^st^, 2^nd^ *etc.*) decreased with the increasing order number. Consistent with 0-order, a slight difference in diffracted intensities (*e.g.* for the 1^st^ order) was also observed between both samples: blue, green and red illumination resulted in 1-order intensities of 3.2, 2.9 and 2.7% for 2.5D grating, and 7.7, 6.8 and 6.2% for the PDMS stamp, respectively. Notice that the light distribution in diffraction depends on the incident wavelength. For shorter wavelengths, lesser transmission to the 0-order meant a stronger diffraction, such that the light was distributed more among the subsequent orders, whereas for longer wavelengths, more light was transmitted to the 0-order without being diffracted. Angle-resolved measurements confirmed that the diffraction angles for original 2.5D grating and PDMS replica were identical. The diffraction angles between normal and 1^st^-order peaks for different lasers, blue, red and green were 10°, 13° and 16°, respectively, consistent with the Bragg's law.

**Fig. 4:**
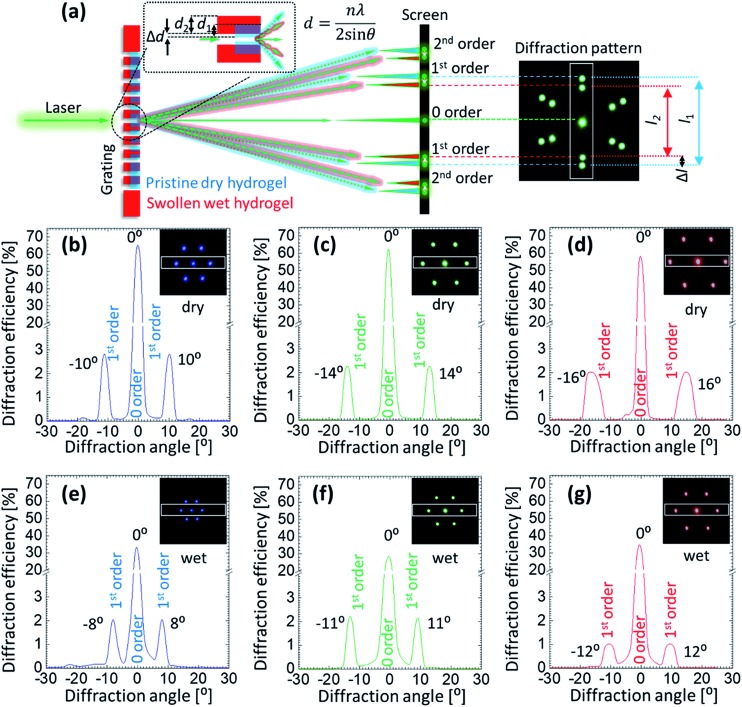
(a) Schematic illustration explaining how the volumetric change of a hydrogel influences the diffraction angle (*θ*) of the transmitted light by changing the groove constant of the microstructure on the surface. (b–g) Angle-resolved intensity graphs of the transmitted light from the GSH in dry and wet conditions. Insets show the photographs of the diffraction patterns taken on the imaging screen. The images were obtained in transmission mode by illuminating the dry and wet samples with blue, green and red lasers, respectively.

Angle-resolved diffraction measurements were carried for the GSH in its dry and wet conditions, Fig. 4. This was done before carrying out the glucose sensing experiment as hydrophobic nature of sensing material's resulted in an initial swelling that needed be taken into account beforehand in order to perform an error-free measurement. When the sample was soaked in PBS, it absorbed the liquid and swelled in all 3 dimensions. During the analysis, two main observations were made in the behavior of diffraction patterns: firstly, the intensity (efficiency) of the transmitted light dropped significantly when the sample was wet. For dry (wet) condition, the efficiency of 0-order spot was 64 (32), 63 (28) and 58% (34%) for blue, green and red lasers, respectively. The decrease in efficiency in the wet condition can be explained by Beer–Lambert law, which states that increasing the thickness of the material in which light is traveling, decreases the light transmission. As soon as the sample underwent the initial swelling as the result of absorbed PBS solution, more light was absorbed by the swollen material. Secondly, the diffraction angle of the transmitted laser light decreased when the sample was in its wet condition. Diffraction angles between 1^st^-order spots generated by the dry (wet) sample were ∼10° (8°), 14° (11°) and 16° (12°) for blue, green and red lasers. By the same token, the distance between 1^st^-order diffraction points projected on the image screen also decreased. The reason for such negative shift of the diffraction angle is the increase in the gap size (groove constant) of the micro-grating imprinted on the hydrogel.

Absorption of PBS by the hydrogel sample resulted in its three-dimensional expansion, thereby, expanded the surface and the features present on the surface. According to the Braggs equation, *nλ* = 2*d* sin *θ*, where, *n* is the diffraction order, *d* is the groove constant, and *θ* is the diffraction angle, the observed shift in the diffraction pattern can be explained. Therefore, volumetric change of the hydrogel material was detected by analyzing the changes in the diffraction pattern generated by the light transmitted through the grating imprinted on the sample. Also, it was established that the resolution of the sensor strongly depended on the wavelength of the laser light that illuminated the sample. Red laser resulted in better resolvable measurements as compared to the shorter wavelengths.

**Fig. 5:**
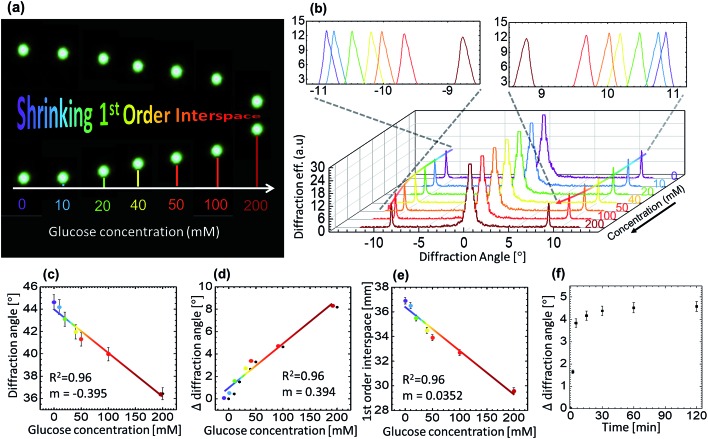
Optical sensing of glucose using GSH sensor: (a) photograph of the interspace between 1^st^-order points changing due to increasing glucose concentration (0-order is masked), (b–d) the change in diffraction angle of the 1^st^-order with increasing glucose concentration, (e) the change in the interspace of the 1^st^-order with increasing glucose concentration, and (f) time-response of the sensor – measurements to record the change in the diffraction angle for 100 mM glucose level with time.

For glucose sensing, angle-resolved diffraction measurements were carried out in far-field by normally illuminating the GSH grating sensor with a green laser and recording the diffraction pattern on an imaging screen located at a distance of 45 cm away from the sensor, see Fig. 5(a) for the snapshots of the 1^st^ order interspace taken for increasing glucose concentration. Increasing glucose concentration can be appreciated by noticing a negative shift in the diffraction angle/1^st^-order interspace resulted by increasing groove constant of the illuminated GSH structure. Such observation is reversible, that is, the diffraction angle increased or decreased due to the shrinking or swelling of the grating upon exposing the same sensor to low or high glucose concentrations, respectively. Fig. 5(b) shows the diffraction efficiency *versus* diffraction angle (between 0 and 1^st^-order) after the sensor was soaked in different solution of different glucose concentrations for 1 h. When the sample was soaked in PBS (without glucose) the diffraction angle was ∼28°. Subsequently, after removing the PBS solution, different glucose solutions were added one by one to examine their effect on the diffraction pattern – the diffraction angle decreased due to the increasing sensor size with a maximum change of ∼8° for 200 mM glucose solutions. In this experiment, the lowest concentration that could be detected accurately was ∼10 mM, for which the change in the 1^st^-order interspace was ∼3 mm (diffraction angle ≈ 0.3°), compared with the PBS-soaked condition. However, this value of sensitivity could be improved considerably by refining various experimental parameters, such as the laser spot size, distance between the GSH and the imaging plate and, using a more precise rotation stage.

Response time is a parameter that determines how fast does the sensor work. It is important because a quick real-time capture of the change in sugar level leads to a better treatment/management. Fig. 5(d) represents the change in the angle over time for the 100 mM glucose concentration solution. Within first 10 min, a rapid change was observed, that moved towards the saturation at ∼15 min, the change after 15 min was negligible. It is important to note that its not only the interspace that could be translated to different concentrations, the time slope for different glucose concentration is also different. Therefore, the change in glucose concentration can also be measured well before 15 min by measuring the slope of the interspace-time curve. Other studies suggest that the sensors may take over 1 h to respond.^42^ In this work, we have demonstrated much faster response time as compared with previous studies. Further improvement in the response time can be achieved by using a thin GSH grating or/and a more responsive phenylboronic acid (however, this is the subject of a separate report).

**Fig. 6:**
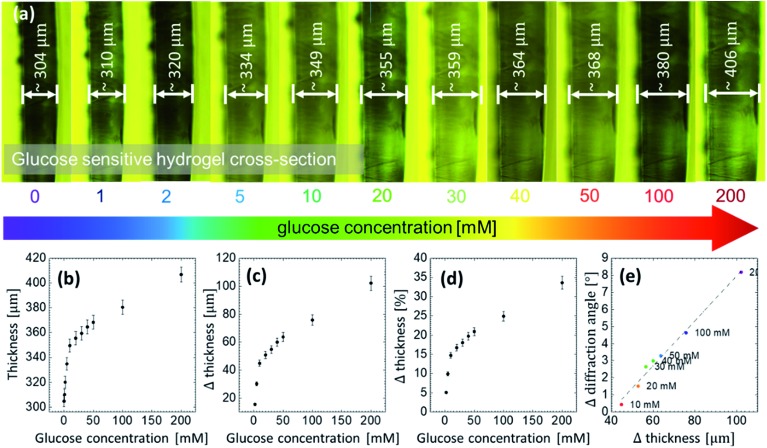
Glucose sensing *via* direct measurements of the dimensions (thickness) of the GSH: (a) the volumetric change of the sample in the presence of glucose at different concentration, (b–d) the change in the thickness for glucose concentration from 0 to 200 mM, and (e) the correlation between the change in the diffraction angle and the change in the thickness.

A thin slice was cut off the GSH sample and placed under the microscope to measure its thickness and its direct response to different glucose concentrations. The slice was placed vertically between two small glass slides and adjusted on a transparent Petri dish. Then, the buffer solution of 7.4 pH was poured into the dish in order to measure the initial increase in the thickness, that is, in the presence of the buffer reference. The initial thickness of the sample without glucose was ∼305 μm. Subsequently, the sample was soaked in different glucose solutions. With increasing glucose concentration, thickness increased, see Fig. 6(a). The lowest detectable glucose concentration was 1 mM. For this concentration, the thickness increased by ∼7 μm, which is ∼2% of the initial thickness. At 200 mM, the thickness increased by ∼34%. A linear correlation was found between the cross-section thickness and glucose concentration at low concentrations, that is, within the range between 1 to 10 mM, see Fig. 6(b–d). Notice that the said range is actually the physiological range and could be useful in sensing application. Extension of this work to measure the blood or urine glucose concentration are the subject our next report. It suggests that the swelling process is uniform in all 3 dimensions: from microscopic images, comparing the change in the thickness in *z*-axis with the change in the *x*–*y* plane extracted from the diffraction angle measurements, a linear correlation between the change in thickness and the change in the diffraction angle is obtained, see Fig. 6(e).

Although the thickness measurements gave a better resolution as compared with the optical measurements due to our experimental limitations below 10 mM, there is considerable room to refine the diffraction experiments for much higher resolution. The difficulties of detecting the change in the diffraction angle for low concentration can be overcome by using small feature size of the diffraction grating, using longer laser wavelength and decreasing the spot size. More responsive (larger swelling coefficient) phenylboronic acids for larger glucose modulated changes in the imprinted patterns can also be used for enhanced sensitivity and improved selectively, such as 2-(acrylamido)phenylboronate, bisboronic acid, and 4-vinylphenylboronic acid.^33^,^43^–^45^ Increasing the surface area by making the nanoporous structures and introducing a gating membrane have also been proven to increase the analyte diffusion and rate of complexation.^46^ Borrowing the similar techniques from previous studies can also help improving the performance of our proposed glucose sensor. Table 1 highlights some of the recent strategies employed to monitor glucose concentration and their challenges as compared to the standard electrochemical method. Optical detection of glucose can lead to an alternative way to the non-invasive, continuous glucose monitoring in a point-of-care setting for diabetic and non-diabetic patients in near future.

**Table 1:** Examples of different types of glucose sensors

Type/work principle		Readout	Range	Advantages	Drawbacks	Ref.
Electrochemical: measures electrochemical potential between electrodes		Voltage *vs.* glucose concentration	10–1000 μM	It is capable of doing single cell measurements, commercial use	Suffers signal bias and day to day drift	14 and 20
Raman spectroscopic detection: probes vibronic properties of the materials		Raman peak shift *vs.* glucose concentration	2–6 mM	Medium range sensitivity	Raman spectroscopy setup needed	47
Holographic 2 and 3D sensors	Based on Bragg scattering	Colorimetric change *vs.* glucose concentration	100 μM to 100 mM	Medium range sensitivity, can be made for non-invasive use	Complex phenylboronic acid synthesis, slower response rate	22, 29 and 48
Photonic crystal						15, 22, 29 and 40
Imprinted 1D		1 order diffraction peak; measures in 1^st^ order interspace (angle/separation) *vs.* glucose concentration		Medium range sensitivity, can be made for non-invasive use easy synthesis	Slower response rate	Present work

## 3. Conclusion

We have demonstrated a new glucose sensor based on a physically patterned glucose responsive hydrogel. The hydrogel was based on poly-acrylamide, *N*,*N*′-methylenebisacrylamide polymerized with a phenylboronic acid, 3-(acrylamido)phenylboronic acid. The patterning was carried out by micro-imprinting of a hexagonal structure from PDMS mirror-replica of a 2.5D honeycomb grating. Sensing was done by carrying out optical diffraction measurements from the patterned hydrogel surface in the presence of different glucose concentration. Glucose binding with phenylboronic acid resulted in physical swelling of the hydrogel, which led to the expansion of the sensor's surface imprinted with micro-patterns. This change in the Bragg diffraction was measured in a far-field transmission configuration. A clear modulation of the 1^st^-order interspace against varying glucose concentration was recorded. Direct observation of glucose-induced swelling of the hydrogel was carried under an optical microscope. A linear relationship between the surface and volume expansions was established. A minimum glucose concentration of 1 mM was successfully recorded suggesting the sensor's usability in physiological conditions. We demonstrated that the fabrication of such sensors is quick and cost-effective as compared to its conventional counterparts, and it is suitable for the mass production.

## 4. Methods

The boronic acid–diol interaction is highly pH-dependent.^43^ For this reason, all measurements were conducted in phosphate-buffered saline (PBS) at a constant pH 7.4. Stock solutions of phosphate-buffered saline were prepared from PBS tablets (ThermoFisher Scientific). A high concentration glucose solution (200 mM) was prepared by dissolving d-glucose (dextrose anhydrous, Science Lab) in the PBS solution. The buffer solution containing glucose was serially diluted with the PBS to prepare various glucose concentrations in the range from 1 to 200 mM. A fresh solution was prepared for each trial and used immediately after their preparation.

## Conflicts of interest

There are no conflicts to declare.
